# Longitudinal Cognitive Decline in a Novel Rodent Model of Cerebral Amyloid Angiopathy Type-1

**DOI:** 10.3390/ijms21072348

**Published:** 2020-03-28

**Authors:** Dominique L. Popescu, William E. Van Nostrand, John K. Robinson

**Affiliations:** 1 Department of Psychology, Stony Brook University, 100 Nicolls Rd, Stony Brook, NY 11794, USA; dominique_popescu@brown.edu; 2 George & Anne Ryan Institute for Neuroscience, University of Rhode Island, 130 Flagg Rd, Kingston, RI 02881, USA; wvannostrand@uri.edu; 3 Department of Psychiatry and Human Behavior, Warren Alpert Medical School of Brown University, 700 Butler, Providence, RI 02906, USA; 4 Department of Biomedical & Pharmaceutical Sciences, University of Rhode Island, 130 Flagg Rd, Kingston, RI 02881, USA; 5 Department of Psychology, University of Rhode Island, 130 Flagg Rd, Kingston, RI 02881, USA

**Keywords:** cerebral amyloid angiopathy, Alzheimer’s disease, rat model, operant testing, radial arm maze, novel object recognition, longitudinal

## Abstract

Cerebral amyloid angiopathy (CAA) is a small vessel disease characterized by β-amyloid (Aβ) accumulation in and around the cerebral blood vessels and capillaries and is highly comorbid with Alzheimer’s disease (AD). Familial forms of CAA result from mutations within the Aβ domain of the amyloid β precursor protein (AβPP). Numerous transgenic mouse models have been generated around expression of human AβPP mutants and used to study cerebral amyloid pathologies. While behavioral deficits have been observed in many AβPP transgenic mouse lines, relative to rats, mice are limited in behavioral expression within specific cognitive domains. Recently, we generated a novel rat model, rTg-DI, which expresses Dutch/Iowa familial CAA Aβ in brain, develops progressive and robust accumulation of cerebral microvascular fibrillar Aβ beginning at 3 months, and mimics many pathological features of the human disease. The novel rTg-DI model provides a unique opportunity to evaluate the severity and forms of cognitive deficits that develop over the emergence and progression of CAA pathology. Here, we present an in-depth, longitudinal study aimed to complete a comprehensive assessment detailing phenotypic disease expression through extensive and sophisticated operant testing. Cohorts of rTg-DI and wild-type (WT) rats underwent operant testing from 6 to 12 months of age. Non-operant behavior was assessed prior to operant training at 4 months and after completion of training at 12 months. By 6 months, rTg-DI animals demonstrated speed–accuracy tradeoffs that later manifested across multiple operant tasks. rTg-DI animals also demonstrated delayed reaction times beginning at 7 months. Although non-operant assessments at 4 and 12 months indicated comparable mobility and balance, rTg-DI showed evidence of slowed environmental interaction. Overall, this suggests a form of sensorimotor slowing is the likely core functional impairment in rTg-DI rats and reflects similar deficits observed in human CAA.

## 1. Introduction

Cerebral amyloid angiopathy (CAA) is a common small vessel disease recognized as the deposition of β-amyloid (Aβ) peptides in and around the cerebral vasculature. In CAA type-2, amyloid deposition occurs in meningeal and cortical vessels, excluding cortical capillaries. The affected larger vessels primarily show thickened walls, Aβ deposits in the tunica media and adventitia, and smooth muscle cell degeneration [1,2,3,4]. On the other hand, CAA type-1 shows Aβ deposition primarily in microvessels and capillaries. The late-onset sporadic form of CAA is most commonly found in aging populations, including most patients with Alzheimer’s disease (AD) [1,3,4,5,6,7,8,9]. Early-onset familial forms of CAA are driven by mutations in the amyloid precursor protein (AβPP). Clinically, cognitive symptoms typically present as impaired perceptual speed and episodic memory and, in severe cases of disease, manifest with seizures and disturbances in consciousness due to lobar intracerebral hemorrhages [10,11,12].

Transgenic animal models have been useful platforms for investigating amyloid pathology and associated symptomology [12,13,14]. Mice have been the leading model organism in AD, CAA and vascular-mediated cognitive impairment and dementia (VCID) research for decades and have yielded important insights. For example, the Tg-SwDI murine model of CAA expresses low levels of human AβPP harboring the Dutch and Iowa familial CAA mutations and develops early-onset, robust accumulation of Aβ in the cerebral microvasculature and notable cognitive impairments on the Barnes Maze spatial learning task [15,16,17,18]. While key insights may be garnered from such models, mice possess distinct limitations, especially in comparison to rat models. Evolutionarily, mice are more distally related to humans than rats, and their smaller brain size and lower white-to-grey matter ratio may introduce practical confounds in experimentation [19,20,21,22,23]. Most importantly, rat models provide the opportunity for considerably more sophisticated and thoroughly validated cognitive analyses.

The rTg-DI transgenic rat model of CAA type-1 has recently been created and described by our group [24]. This rTg-DI model of CAA demonstrates early-onset, progressive accumulation of cerebral microvascular fibrillar amyloid, robust microhemorrhages with small vessel occlusions, perivascular glial activation and capillary structural changes [24,25]. Measurable functional consequences have been previously observed in this model [24]. However, no longitudinal phenotypic characterization had been performed. Therefore, for the first time, the current study aimed to characterize nuanced changes in behavior by using a comprehensive and progressive operant battery of assessments, tasks based on lever responses to light cues (see methods for detailed descriptions of tasks), to determine the impact of progressive microvascular CAA [18,26,27]. Our analyses showed the rats remained physically healthy throughout the study and could effectively learn the operant procedural requirements, similar to the wild-type (WT) rats. However, rTg-DI rats responded more slowly and, therefore, consistently made fewer responses across multiple tasks as compared to WT rats. These findings are similar to the deteriorating executive function observed in human CAA and underscore the utility of the rTg-DI rat as a preclinical model to investigate the pathogenesis and VCID of this disease.

## 2. Results

### 2.1. Overall Study Design

**Figure 1:**
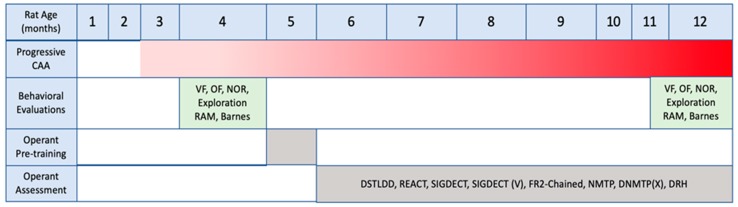
Overview of the study timeline. Rats began non-operant evaluation at ~3 months while pathology first emerged. Then, the rats were habituated to the operant chambers and core responding principles beginning at ~4 months, followed by detailed operant assessments from ~6 into 12 months while pathology continued progressing. Lastly, rats underwent a second round of non-operant evaluations beginning at ~10 months of age before euthanasia at ~12 months. See text for definitions.

**Figure 2:**
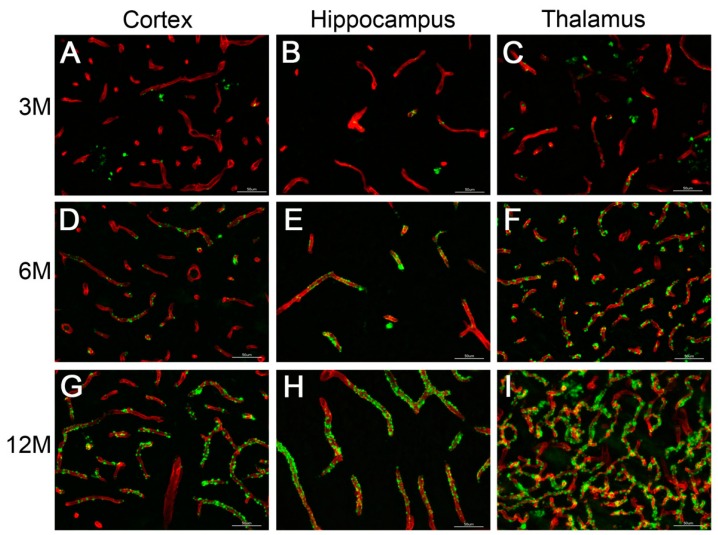
Cerebral microvascular amyloid accumulation in rTg-DI rats. Representative brain sections from 3-, 6- and 12-month-old rTg-DI rats not tested in behavior that were stained with thioflavin S to identify fibrillar microvascular amyloid (green) and immunolabeled with an antibody to collagen IV to identify cerebral blood vessels (red). Progressive accumulation of microvascular amyloid deposits is observed in the cortex (**A**,**D**,**G**) hippocampus (**B**,**E**,**H**) and thalamus (**C**,**F**,**I**). Scale bars = 50 µm.

A timeline of the experiment is shown in Figure 1. Rats were first evaluated in a number of behavioral tasks at 3 months, an age when microvascular amyloid accumulation first emerges in rTg-DI rats, see Figure 2. After this initial behavioral assessment, the rats were habituated to operant training and then began operant assessment at ~6 months, when microvascular amyloid becomes more extensive, see Figure 2 and continued for ~4 months followed by another round of behavioral assessments until the animals were euthanized at ~12 months, when microvascular CAA is severe, see Figure 2. This design allowed us to determine the impact of progressing microvascular CAA, from moderate (~6 months) to severe stages of disease, on the ability of the rats to perform operant tasks.

### 2.2. rTg-DI Rats

rTg-DI rats are a novel transgenic model of cerebral microvascular CAA type-1. These rats produce familial CAA mutant Dutch E22Q/Iowa D23N chimeric Aβ in the brain and begin to develop cerebral microvascular amyloid deposition starting at ~3 months of age [24,25]. CAA severity progresses as the rTg-DI rats age from 6 to 12 months of age, showing extensive microvascular amyloid accumulation in the cortex, hippocampus and thalamus (Figure 2).

### 2.3. Health and Development of Rats

Overall, both rTg-DI and WT rats appeared healthy throughout the course of the study and gained weight as they aged. In both groups, females consistently weighed less than males [F(1,15) = 335.43, *p* < 0.001] and rTg-DI males weighed less than WT males at 10 months, [t(16) = 2.75, *p* = 0.014], 11 months, [t(16) = 3.99, *p* = 0.001], and 12 months of age, [t(16) = 3.36, *p* = 0.004] (see Supplementary Materials Figure S1).

### 2.4. Operant Schedule Assessment

Here, we describe the first longitudinal phenotypic characterization of the rTg-DI rat model of CAA type-1 through extensive operant assessment and non-operant cognitive and physical evaluations, see Table 1 for task summary. This detailed and labor-intensive approach offers many advantages over the exclusive use of maze paradigms including the examination of multiple performance measures against stable behavioral baselines, redeterminations of performance on tasks across time points, and an apparatus enclosed in sound-attenuating chambers to reduce ambient confounds. This comprehensive operant approach is rare in the rodent transgenic model field, where brief high-throughput behavioral assays have dominated, and simplistic and potentially misleading conclusions about the behavioral phenotype of rodent lines are often produced.

The light–dark discrimination task, reaction time and signal detection tasks were all based upon a shared visually discriminated operant rule. Accurate responding in these tasks reflected proficient discrimination learning. The rate of reward was maximized by accurate discrimination along with high rates of trial completion. The latter tasks (reaction time and signal detection) added more stringent response criteria or stimulus discriminability challenges to this basic cue-response-reinforcement contingency. Together, this approach allowed us to assess fundamental functions before adding more challenging conditions, as well as to return to baseline conditions to quantify loss of function as a result of underlying disease progression.

All analyses held to *p* < 0.05 standard. One-way analyses of variance (ANOVA) were performed to compare groups at a single time point. Comparisons across time points were carried out by repeated measures, or within-subjects, ANOVA, unless otherwise specified. If the sphericity assumption was violated, the degrees of freedom of the *F*-distribution were adjusted by the Greenhouse-Geisser correction, and multiple comparisons were adjusted for by the Bonferroni correction.

#### 2.4.1. Light–Dark Discrimination

**Figure 3:**
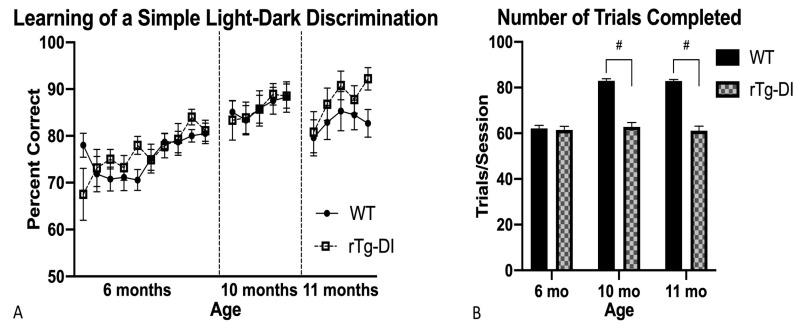
Learning and response activity in a simple light–dark discrimination task (DSTLDD). (**A**) Learning of the DSTLDD rule: accuracy increased across initial acquisition trials at 6 months and redeterminations, which occurred at 10 and 11 months of age; initial accuracy improved across trial days at 6 months and responses were more accurate at 10 months. Overall, rTg-DI responses were as accurate as WT. (**B**) DSTLDD average trials completed: rats responded as actively and therefore completed as many trials at 6 months; however, at 10 months rTg-DI responded less than WT and this pattern of diminished responding persisted at 11 months. # *p* < 0.05. Data represent mean + SEM.

Both groups learned the discrimination task comparably during initial training at 6 months, retesting at 10 months, and again at 11 months of age, and rTg-DI rats responded as accurately as WT overall at all time points (Figure 3A). However, while animals completed a similar number of trials at 6 months of age, rTg-DI completed fewer trials at 10 months [*F*(1,19) = 6.34, *p* = 0.021] than WT and again at 11 months [*F*(1,19) = 6.3, *p*= 0.022], indicating a potential limit in response activity since the WT rats showed a clear training effect at the later time points (Figure 3B). In all, rats successfully demonstrated acquisition and retention of this discrimination rule, though rTg-DI rats responded less actively than WT beginning at 10 months of age. This suggested a developing speed–accuracy tradeoff.

#### 2.4.2. Reaction Time

**Figure 4:**
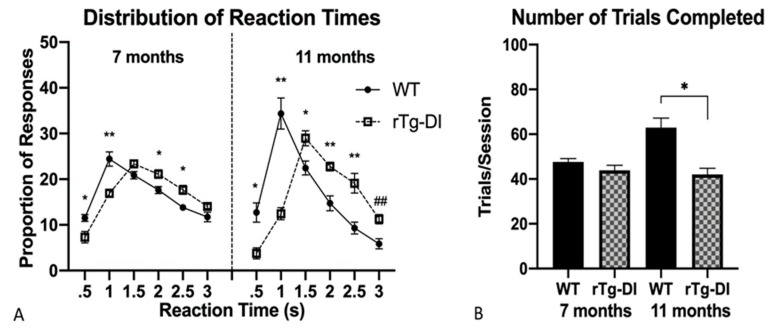
Responses on a reaction time task (REACT). (**A**) rTg-DI rats showed a shifted reaction time distribution at 7 months of age, becoming more pronounced at 11 months. (**B**) rTg-DI rats completed fewer trials than WT only at 11 months of age. Data represent mean +/− SEM. ## *p* < 0.005, * *p* < 0.01, ** *p* < 0.001.

This task was based upon the same basic light–dark discrimination but required the rats to respond on the cued lever within a constrained time (3 s). The frequency of responses within 0.5 s interval bins from cue light onset showed a rightward shift at 7 months and then in a more pronounced manner at 11 months. The rTg-DI rats made a smaller proportion of responses within 0.5 s following cue light onset [*F*(1,19) = 8.97, *p* = 0.008] and within 0.5–1 s [*F*(1,19) = 15.37, *p* = 0.001] at 7 months despite completing as many trials as WT rats overall. Again at 11 months, rTg-DI made a smaller proportion of responses within 0.5 s of cue light onset [*F*(1,19) = 11.98, *p* = 0.003] and within 0.5–1 s [*F*(1,19) = 30.6, *p* < 0.001] as compared to WT (Figure 4A). However, rTg-DI rats at 11 months responded less actively by completing fewer trials [*F*(1,19) = 9.14, *p* = 0.007] than WT (Figure 4B). The shift in rTg-DI response distribution towards slower reaction times represents subtle response initiation slowing that emerged when a high demand to respond quickly was introduced; this preceded the speed–accuracy tradeoff evident at 11 months in reaction times and at 10 and 11 months in light–dark discrimination.

#### 2.4.3. Signal Detection

The ability to detect and initiate motor responses to cue light in this task was measured by percent correct responses to each signal duration, and, again, response activity was measured through total responses completed and omitted responses. This task was based upon the same basic light–dark discrimination task but shortened the duration of the discriminative stimulus to 100, 300 or 1000 ms, thereby requiring sustained attention to the front panel in order to discriminate successfully. At 7 months, the rTg-DI rats responded as accurately to varying signal durations as WT animals at early and late learning time points. Response accuracy improved with increasing signal duration in both groups, confirming that the shorter duration stimuli were more difficult to detect [*F*(2,36) = 12.36, *p* < 0.001]. Interestingly, in this task, rTg-DI rats were not significantly less likely to respond than WT rats (see Supplementary Materials Figure S2). However, they made fewer anticipatory responses that occurred in the 3 s prior to the stimulus presentation than WT (rTg-DI *M* = 23.1, *SD* = 11.1; WT *M* = 36.8, *SD* = 11.6; *F*(1,19) = 7.18, *p* = 0.015). Therefore, while, overall, the rTg-DI rats performed comparably to WT on this main signal detection measure, the slower reaction times of the rTg-DI observed on the previous reaction time task may have manifested here as fewer anticipatory responses rather than reduced trial completion.

#### 2.4.4. Signal Detection, Variable Pre-Stimulus Interval

This task differed from the previous signal detection task in that the pre-stimulus interval varied from the fixed 10 s to either 1, 3 or 10 s, presented randomly. This increased the density of the trials and might have revealed a decreased response rate in the rTg-DI rats at 8 months. Again, and in contrast to the early discrimination tasks, both groups responded with similar accuracy and frequency (see Supplementary Materials Figure S3) and omitted a similar number of trials to the 1000 ms duration. However, rTg-DI (*M* = 18.92, *SD* = 12.02), again, made fewer anticipatory responses than WT (*M* = 33.38, *SD* = 13.04) [*F*(1,19) = 6.52, *p* = 0.02].

#### 2.4.5. FR2-Chained Responding

This task and the non-matching tasks that followed employed a more complex conditional discrimination rule, where the correct discriminative response was based upon the position of a previous cue or cues and responses. The FR2-chained task introduced the rear lever and cue light, requiring rats to travel to the back of the operant box and respond on the cued rear lever prior to responding on one of the cued front levers. At 9 months, rats from both groups responded with similar accuracy and completed as many trials on the initial days of testing. However, on later days of testing, the rTg-DI rats responded more accurately than WT [*F*(1,19) = 5.0, *p* = 0.038] despite completing fewer trials overall [*F*(1, 19) = 6.8, *p* = 0.018] (see Supplementary Materials Figure S4).

#### 2.4.6. Non-Matching to Position (NMTP)

**Figure 5:**
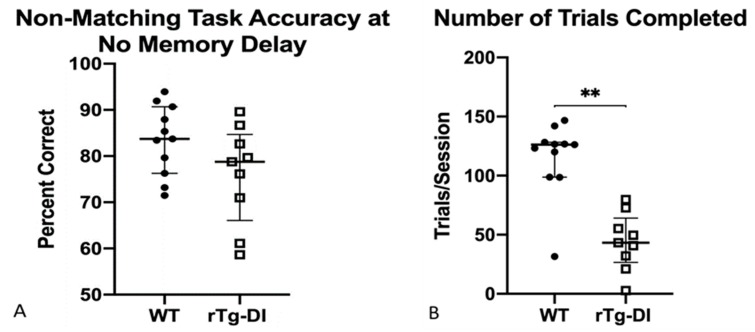
Learning and responding on a non-matching response task without an added memory delay (NMTP) at 9 months of age, WT data represented as filled circles, rTg-DI represented as boxes. The previously-seen pattern of accurate responding (**A**) offset by fewer responses (**B**) is evident again on NMTP. ** *p* < 0.001. Data are represented as statistical dispersion or median averaged across number of trials to meet criteria + interquartile range.

This task requires a sequence of three responses to receive a reinforcer. Initially, one of the front cue lamps was illuminated and a response was required (the ‘sample position’). Next, as in FR2, the rear cue lamp was illuminated, and a response was again required. Finally, a response was required on the opposite front lever from the one first pressed during the sample (the ‘non-match’ response). Importantly, both cue lamps are illuminated for the non-match response phase, so that the rat must remember the sample position cue to perform accurately. Both groups of rats responded to NMTP with similar accuracy at the end of the training period (Figure 5A). However, rTg-DI responded less frequently [*F*(1,19) = 8.32, *p* = 0.01].

#### 2.4.7. Delayed Non-Matching to Position: 5 s Delay Time Point, DNMTP (5)

**Figure 6:**
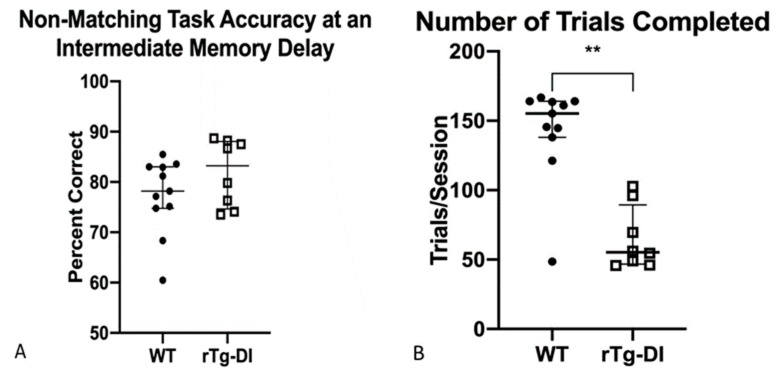
Accuracy and frequency of responding on a non-matching response task at a moderate 5 s memory delay added (DNMTP) at 10 months of age, WT data represented as filled circles, rTg-DI represented as boxes. rTg-DI rats responded as accurately as WT rats (**A**) but completed fewer sequences than WT rats (**B**) ** *p* < 0.001. Data are represented as statistical dispersion or median across number of trials to meet criteria at the 5 s delay + interquartile range.

This variation of the NMTP task introduced a variable delay before final NMTP response was required and, therefore, increased the short-term memory demand. Summary data are presented from the intermediate 5 s delay condition at 10 months of age. Once again, both groups responded with similar accuracy and rTg-DI completed fewer responses than WT [*F*(1, 18) = 31.2, *p* < 0.001], (Figure 6A,B).

#### 2.4.8. Differential Reinforcement: High Responding (DRH)

**Figure 7:**
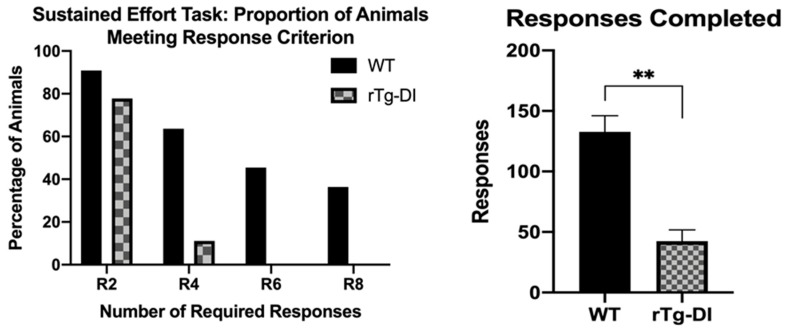
The number of rats to successfully meet response criteria and total responses completed on a sustained effort task (DRH) at 11 months of age. (**A**) rTg-DI met a lower response criterion (two responses within 10 s in the R2 condition, four responses within 10 s in the R4 condition, and so on) than WT rats. (**B**) DRH average responses; rTg-DI made fewer overall responses during the R2 condition than WT rats. ** *p* < 0.001. Data represent mean + SEM.

This task differed from all the others used previously, in that it required rats to simply complete a series of responses on a single lever within a particular time interval to receive reinforcement. Therefore, it served to emphasize the ability of the rat to respond with a series of responses in a constrained period of time. At 11 months, the rTg-DI rats were strikingly different than WT in that they made fewer responses [*F*(1,19) = 27.96, *p* < 0.001] (Figure 7B) than WT rats, resulting in most rTg-DI rats only successfully meeting the minimal two responses/10 s DRH response criteria level (Mann–Whitney non-parametric test U = 22, *p* = 0.028; Figure 7A).

### 2.5. Non-Operant Behavioral Evaluation

#### 2.5.1. Open Field

**Figure 8:**
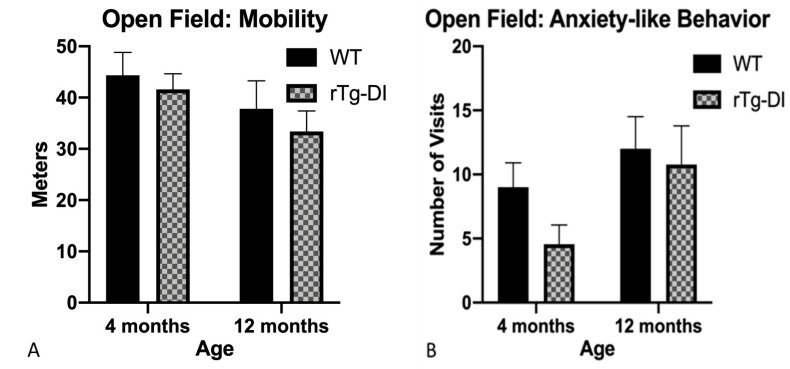
Exploration of the open field. (**A**) Mobility was measured by the average distance traveled and (**B**) anxiety-like behavior as measured by average number of entries to center region of the open field. Data represent mean + SEM.

At 4 months, both groups were similarly active and demonstrated similar levels of anxiety-like behavior. At 12 months, the exploration patterns of both groups were comparable (Figure 8A,B).

#### 2.5.2. Radial Arm Maze

**Figure 9:**
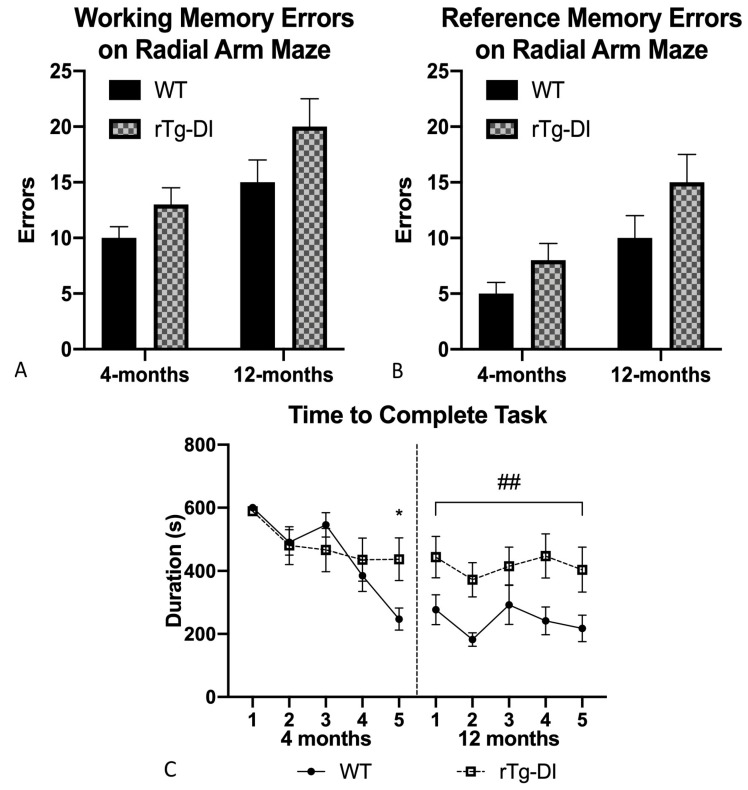
Radial arm maze. (**A**) Average working (WME) and (**B**) reference memory errors (RME) across 5 trial days were only marginally elevated in the rTg-DI rats though rTg-DI took longer to complete trials at 12 months than WT rats (**C**) ## *p* < 0.005, * *p* < 0.01. Data represent mean + SEM.

In this task, the rats explored an array of arms emanating from a central zone. Five of those arms contained a reinforcer at the end and three never contained a reinforcer. Revisiting an arm at which the reinforcer had previously been consumed defined a working memory error and entering an arm that never contained a reinforcer defined a reference memory error. At 4 months, rTg-DI rats completed sessions as quickly, though time to complete this task differed on the last trial day as rTg-DI took longer to consume all rewards. The rTg-DI rats made as many reference and working memory errors as WT rats (Figure 9A,B). However, by 12 months, differences in task duration [*F*(1,17)= 14.056, *p* = 0.002] emerged, as rTg-DI rats look longer than WT rats to complete the task (Figure 9C). Both groups made a similar number of working memory errors at the 12-month time point (Figure 9A), though rTg-DI rats made marginally more reference memory errors [*F*(1,17) = 4.363, *p* = 0.052] than WT rats. An age-related increase is evident for both groups.

#### 2.5.3. Novel Object Recognition (NOR)

**Figure 10:**
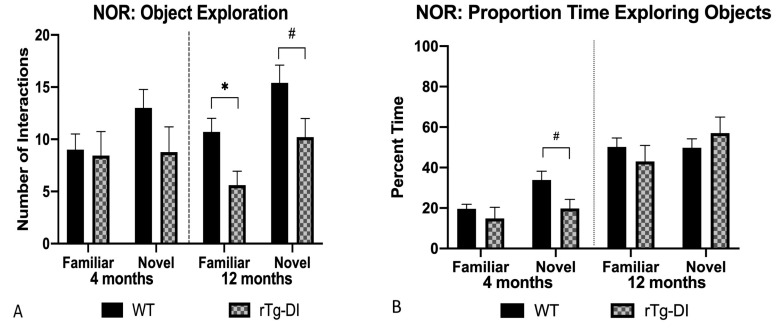
Novel object recognition. (**A**) rTg-DI rats had fewer interactions at 12 months with both the familiar and novel objects than WT. (**B**) rTg-DI spent a smaller proportion of time interacting with the novel object at 4- but not 12-months than WT rats. # *p* < 0.05*,* * *p* < 0.01. Data represent mean + SEM.

In the NOR task, the rats were placed in the open field and allowed to interact with two objects for 10 min. On a second day, one of the same, familiar objects from the previous day and one novel object were available for exploration. Preference for the novel object is thought to reflect intact memory capacity. At 4 months, the exploration patterns of both groups were not significantly different, though rTg-DI rats appeared to not show any preference for the novel object (Figure 10B). However, at 12 months, rTg-DI rats had fewer interactions with both the novel object [*F*(1,18)= 4.46, *p* = 0.05] and with the familiar object [*F*(1,18) = 9.97, *p* = 0.006] than WT (Figure 10A). This suggests slowed exploration rather than deficient memory capacity, and evidence for slowed exploration was also observed on a novel exploration task at 12 months. Confirming this, when presented with an array of four novel objects, rTg-DI made fewer interactions with two of the objects (*M* = 7.13, *SD* = 2.3, *M* = 5, *SD* = 3.12) compared to WT rats (*M* = 10, *SD* = 2.72, *M*= 8.45, *SD* = 3.59).

#### 2.5.4. Barnes Maze

In this task, the rats explored an exposed circular arena with an array of holes around the periphery. One hole had a box secured underneath it into which the animal could escape. At 4 months, performance improved on this task across trial days though no difference was evident across the groups on latency to escape, path efficiency, and visits to error hole locations. At 12 months, a comparably quick reacquisition was evident in both groups across 2 trial days, and path efficiency and visits to error hole locations remained similar (see Supplementary Materials Figure S5).

#### 2.5.5. Von Frey Hairs

This method is used to assess paw withdrawal reflex to tactile stimulus. The rats were placed on an enclosed platform that allowed access to the plantar surface of their paws. Von Frey filaments of increasing thickness were pressed to the paws and a sign of paw withdrawal was noted, with responses to thicker hairs indicative of a higher sensitivity threshold. A Mann-Whitney non-parametric test indicated front and back paw sensitivity levels were similar between rats at 4 months. However, at 12 months, a moderate decrease in rTg-DI front paw (rTg-DI Mdn = 5.0 vs. WT Mdn = 4.47; U = 22.5, *p* < 0.05) and back paw sensitivity (rTg-DI Mdn = 5.18 vs. WT Mdn = 5.07; U = 23.5, *p* < 0.05) was observed, see Supplementary Materials Figure S6.

### 2.6. CAA Pathology

**Figure 11:**
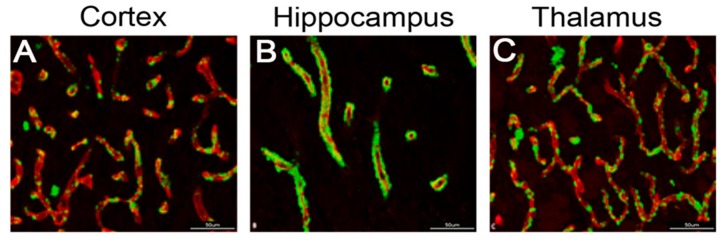
Cerebral microvascular amyloid accumulation in rTg-DI rats was unaffected by operant training. Representative brain sections from 12-month-old rTg-DI rats at the completion of operant training and behavioral analysis that were stained with thioflavin S to identify fibrillar microvascular amyloid (green) and immunolabeled with an antibody to collagen IV to identify cerebral blood vessels (red). Robust microvascular amyloid deposits are observed in the cortex (**A**) hippocampus (**B**) and thalamus (**C**). Scale bars = 50 µm.

At the conclusion of the operant training protocol and behavioral analysis at 12 months, the rTg-DI rats showed evidence of extensive cerebral microvascular amyloid deposition in the cortex, hippocampus and thalamus (Figure 11A–C respectively). This pattern of microvascular amyloid deposition is essentially the same as observed in rTg-DI rats that underwent no training, see Figure 2, indicating that the extensive operant training itself did not alter the nature of the CAA pathology in the rats.

## 3. Discussion

CAA is a common cerebral small vessel disease, frequently observed in AD, which promotes VCID. Recently, we developed the rTg-DI rat model of microvascular CAA type-1 [24,25]. Here, for the first time, we report on the detailed longitudinal behavioral characterization of the novel rTg-DI model through physical and cognitive evaluations and detailed operant assessments. Our aim was to gain new insight into the unique cognitive impacts of microvascular CAA.

The rTg-DI rat model develops early-onset and progressive accumulation of Aβ in and around the cerebral microvasculature, which promotes structural changes in capillaries and perivascular inflammation, robust microhemorrhages, and small vessel occlusions [24,25]. A characterization of pathology over the course of 3 to 12 months was previously completed [24,25]. At 3 months, notable accumulation of cerebral microvascular Aβ in cortical, thalamic and hippocampal regions was previously reported. While this was the age of onset of Aβ deposition in the forebrain, the composition of these deposits at 3 months formed a consistent pattern that was more extensive at 6 and 12 months. Insoluble Aβ40 comprised the largest proportion of forebrain deposits, followed by comparatively modest levels of insoluble Aβ42. At 6 months, capillary accumulation of amyloid progressed. Although no evidence of microbleeds in the cortex or hippocampus was reported at 6 months, clear evidence of microbleeds was detected in the thalamus. Additionally, small thalamic vessels in the ventral posterior lateral nucleus (VPL), surrounded by fibrinoid necrotic areas, showed evidence of occlusion and calcification. By 12 months, robust accumulation of cerebral microvascular Aβ was detected. Although some cerebral microbleeds were found in the cortex and hippocampus, the thalamus was particularly impacted by microbleeds. Further, increases in perivascular reactive astrocytes and activated microglia, inflammatory marker expression and loss of perivascular pericytes were observed [24,25].

### Progression and Nature of the Behavioral Impairment

Physical evaluations at 4 months of age indicated the rTg-DI rats were generally healthy and demonstrated similar basic motor abilities as WT. Rats actively explored their environment, responded to tactile stimuli, and maintained bodyweight throughout the study. However, sex-specific differences in bodyweight were noted, and, as expected, males outweighed females regardless of genotype. In addition, cognitive evaluations at this time showed no working and reference memory impairments but revealed differences in object interactions. Here, rTg-DI completed the radial arm maze (RAM) and Barnes maze efficiently, making as many error arm or hole visits as WT rats. However, when presented with a previously encountered and a novel object simultaneously within a familiar context, rTg-DI rats interacted with the novel object less than WT; However, the rTg-DI rats spent a similar proportion of time with the paired objects, overall. This differing pattern of object interaction was also observed on a novel exploration task; interactions were observed with multiple novel objects within a familiar context.

In the operant battery, a consistent pattern of difference between the rTg-DI and WT emerged. On many tasks, especially the later redeterminations of several procedures, the rTg-DI rats efficiently expressed the learned task rules (reference memory) by maintaining comparable or even superior levels of accuracy. Notably, however, they often completed fewer trials per session than WT rats. This speed–accuracy tradeoff was observable as early as 7 months. An especially striking variation of this impairment was evident on the DRH task. Despite the apparent simplicity of the task demands (X responses within 10 s), the requirement of completing the fixed ratio quickly appeared daunting to the CAA rats. Most rTg-DI rats could not complete DRH4, a modest rate requirement of one response every 2.5 s. In short, rTg-DI rats appear to sacrifice speed to complete the task for higher accuracy in performance.

The non-operant assessments at 12 months affirm this trend. Cognitive assessments at this time point indicated that rTg-DI rats interacted with their environments more hesitantly. rTg-DI rats explored at a slower pace through the RAM compared to WT at 12 months. rTg-DI rats took longer to complete the RAM and Barnes mazes while making marginally more reference memory errors yet completed the Barnes maze as efficiently. Furthermore, rTg-DI rats had fewer object interactions than WT on NOR and dedicated a smaller proportion of time to interacting with the set novel objects on a novel exploration task than WT rats.

rTg-DI rats have reportedly shown differences in revisits to objects when presented with a set of four novel objects at 3 months, compared to age-matched controls [24], and increased latency to escape the Barnes maze has been reported as early as 3 months of age in the murine Tg-SwDI model [16]. Taken together, the differences measured by the 4-month evaluations complement these previous findings. The timeline of measured pathology development, followed by observed behavioral differences, aligns with the understanding of disease development and symptom expression in humans, as pathology typically develops well before the onset of symptom expression. Additionally, a progressive cognitive stimulation operant assessment was previously completed with the murine Tg-SwDI model [27]. In this case, Tg-SwDI mice successfully completed as many operant tasks as WT controls including light–dark discrimination, FR2 response contingency discrimination, non-matching to position and delayed non-matching to position, as measured by the number of sessions to meet response accuracy criteria. Interestingly, Tg-SwDI mice met response accuracy criteria when tasked with longer retention intervals on delayed non-matching to position at 6 months than WT controls, though this delay interval is comparatively shorter than the intervals rTg-DI rats successfully completed [27]. Taken together, the current findings surrounding progressing behavioral deficits and the equally important functional sparing in the rTg-DI rats support previous findings. Ultimately, the progression of thalamic pathology at this point may have impacted rTg-DI motor initiation or execution and as thalamic vessel occlusions and calcifications were not previously observed in the Tg-SwDI murine model the differences in response ability may pertain specifically to thalamic pathology in the rTg-DI rat model. By 12 months, rTg-DI rats reportedly showed differences in revisits to objects when presented with a set of four novel objects at 12-months, compared to age-matched controls [24], and the murine Tg-SwDI model took longer to escape the Barnes circular maze as compared to age-matched controls. Differences in object interactions on NOR were also noted in the murine Tg-SwDI as compared to WT mice [16]. Following 4 months of progressive cognitive stimulation operant training, the Tg-SwDI murine model showed evidence of task acquisition on Barnes circular maze, however, despite months of training, still took longer to escape than WT mice. Furthermore, this previous study found that naïve, control and progressively trained Tg-SwDI mice took similarly longer to escape and expressed similar levels of cerebral insoluble Aβ despite the training condition [27].

These impairments contrast somewhat with the impairments reported in transgenic rat AD models. An exact comparison is difficult, as many of the behavioral validation methods for these rat AD models rely almost exclusively on maze tasks, including the Morris water maze, a task we chose not to employ, and have never employed the comprehensive operant testing battery used presently. However, spatial episodic memory impairment was reported in the PSAPP [28], McGill-R-Thy1-APP [29] and TgF344-AD [30] rat lines and has usually been interpreted in terms of selective hippocampal damage. Our current data show no compelling evidence of working or short-term memory impairment in DNMTP, Barnes maze, or NOR tasks, illustrating non-overlapping effects between these AD models and our CAA rTg-DI rats and pointing again to the importance of compromise in thalamo-cortical circuits rather than hippocampal dysfunction in this model. Indeed, our data are more consistent with a cholinergic-dependent, thalamo-cortical dysfunction, which would disturb sensorimotor integration and whose disruption may underlie memory and other deficits across a variety of neurological disorders, including AD and Parkinson’s disease [31,32,33,34]. While our data may also reflect two or more distinct impairments, the two kinds of behavioral data considered parsimoniously are consistent with a generalized ‘cognitive slowing’ slowing effect as primary. This slowing could result in both more effort required to maintain accurate performance in operant tests and slower rates of stimulus integration in the non-operant tests. This ‘cognitive slowing’ observed in rTg-DI rats is not inconsistent with sensorimotor and perceptual slowing deficits reported in CAA patients [35,36,37], though various reports have also implicated episodic memory, visuospatial and executive function deficits also present in CAA [38,39,40]. However, the sensorimotor integration/perceptual slowing observed presently still underscores the utility of rTg-DI rats as a preclinical model to further investigate the pathogenesis of CAA and associated VCID. For example, while the present study was underpowered to address potential sex differences in the onset and severity of behavioral symptoms, this is an interesting question where future studies will be needed to address this pertinent topic directly through study design, as well as the potential contributions of various environmental factors in preventing or exacerbating pathology and behavioral deficits.

## 4. Materials and Methods

### 4.1. Subjects

This longitudinal study included WT (*n* = 11), male (*n* = 5) and female (*n* = 6), and rTg-DI (*n* = 9), male (*n* = 5) and female (*n* = 4), rats. The rTg-DI animals were created on a Sprague–Dawley background and express human amyloid β precursor protein (APP) gene containing the Swedish *K670N/M671L*, Dutch *E693Q* and Iowa *D694N* mutations under the control of a Thy1.2 promoter. The creation and characterization of this line was previously reported [24]. Non-transgenic littermates from rTg-DI breedings served as WT controls. One rTg-DI rat was euthanized prior to the conclusion of the study; therefore, those data were excluded from the endpoint evaluation analyses.

The rats were housed in a controlled room (22 ± 2 °C and 40–60% humidity) on a standard 12 h light on cycle. The rats were allowed to habituate to the controlled room environment for a week prior to the start of the study, and then baseline evaluations began at 4 months of age. Rat chow was available *ad libitum*, and body weights were recorded weekly throughout this study. All animal experiments were approved by the Institutional Animal Care and Use Committee at Stony Brook University and conducted in accordance with the United States Public Health Service’s Policy on Humane Care and Use of Laboratory Animals.

### 4.2. Apparatus and Testing Procedures

Operant chambers (MED Associates, Fairfax, VT, USA), measuring 30.5 cm × 24.1 cm × 21.0 cm were contained inside a sound attenuating chamber. An exhaust fan provided white noise within the chamber. A front panel contained two front levers, one each to the right and left of the water delivery mechanism. A cue lamp was positioned over each lever. One additional response lever was centered on the rear chamber wall, also under a cue lamp. The rats were not allowed access to water in the home cage for 23 h prior to the testing sessions, though animals were allowed 1 h of free water access to water after each session and free water access on weekends. A 0.1 mL drop of water delivered by a solenoid served as the reinforcer for all tasks. All detailed operant events and measures were controlled by MED-PC software.

### 4.3. Operant Schedule Training

5–11 months of age: Pre-assessment training began at 5 months of age and consisted of simple testing environment habituation sessions, a lever response-reinforcement association and an alternating lever response task, see Figure 1 for task order. Typically, animals underwent one 30 min session per day for a maximum of 5 consecutive days. Operant testing began with a light–dark discrimination task once each rat’s response accuracy exceeded 80% correct on an alternating response position task. The endpoint behavioral assessment began at 11.5 months, during which an operant testing schedule was maintained until 12 months of age, although no rat completed both operant and behavioral evaluations on the same trial day. As much of the behavioral testing methodology has been previously described by our research group [18,24,25,26,27], overlapping procedures will be described here briefly.

### 4.4. Testing Environment Habituation and Pre-Training

Rats were habituated to the operant chamber and associated the sound of the solenoid reinforcement delivery mechanism and access to the reinforcement itself. Initially, water reinforcement delivery occurred every 15 s, non-contingently. If responses occurred on either front lever reinforcements were delivered. Next, the rats were introduced to the relationship between lever responses and reinforcement delivery. A response on either the right or left lever elicited reinforcement delivery. Finally, the rats were required to alternate responses on the right and left levers for reinforcement.

### 4.5. Operant Schedule Assessment Procedures

6- to 11.5-months of age: Operant assessment began at 6 months of age, following successful completion of all pre- training sessions. All rats underwent a comprehensive, progressive operant training assessment. No rat advanced to the following task until successful acquisition of the previous task. The task advancement criterion was determined either by a total number of responses or a minimum response accuracy level per 30 min trial. Sessions terminated after 30 min expired. Rats were typically tested on 5 consecutive days per week.

#### 4.5.1. Light–Dark Discrimination (DSTLDD)

The illuminated cue light was first introduced on this task. Responses on the lever indicated by the cue to produce reinforcement, illuminated with a 50% likelihood in the left or right position. A 5 s inter-trial interval (ITI) separated the trials.

#### 4.5.2. Response Reaction Time (REACT)

This task required animals to respond on the cued lever within a 3 s time interval from illumination of the cue lamp at the left or right position with a 50% likelihood. Responses were recorded in 0.5 s bins. The ITI was randomly presented as 5, 7.5, 10, 12.5 or 15 s. A failure to respond within the 3 s hold period was an omitted trial, whereas a response prior to cue light onset was scored as an inter-trial response (ITI).

#### 4.5.3. Stimulus Detection (SIGDECT)

This task manipulated the duration of the cue light signal. A trial consisted of a brief orienting tone then a cue light randomly illuminated for 100, 300 or 1000 ms, at 50% probability in the left or right position 3 s after tone offset. Responses were considered omitted when the response occurred more than 5 s following cue light offset. A 10 s ITI separated each trial. Sessions terminated after 30 min.

#### 4.5.4. Signal Detection Varied Pre-Stimulus Interval (SIGDECTV)

This variation on the SIGDECT task introduced a randomly selected interval between the beginning of the trial and the orienting tone. This time interval, previously held constant at 3 s, now varied between 1, 5 and 10 s. The rats were required to remain vigilant for the interval following trial start yet before the cue light in order to successfully complete this variation. The duration of the signal still randomly illuminated for 100, 300 or 1000 ms, as in the previous version of this task.

#### 4.5.5. FR2-Chained Response

This was the first task that employed a more complex conditional discrimination rule, where the correct discriminated response was based upon the position of a previous cue and responses. This task added cued responding on the rear lever in addition to the front right and left levers, requiring the animal to shuttle between the left lever-rear lever-right lever-rear lever-etc. This established the conditional discrimination pattern of front-rear-opposite front lever responding that forms the basis of the non-matching to position task employed later. Trials began with the illumination of one of the front cue lights, and, if the initial response was correct, reinforcement was delivered. Then, the rear cue light was illuminated. A rear lever response was required, and the front cue lamp opposite to that previously illuminated immediately following rear response. A correct response on the front lever produced reinforcement and illuminated the rear cue lamp again. A response on the rear lever was necessary for reinforcement, but not itself reinforced, and unconstrained by a trial response time (hence the “FR2” requirement). A 10-day period in which no training occurred separated trials 6 and 7.

#### 4.5.6. Non-Matching to Position (NMTP)

This task included responding on all levers, similar to the previous FR2-chained response task, and introduced a short-term memory-dependent rule requiring a lever response, following the rear response, on the opposite lever than initially cued. The lever response sequence mirrored the FR2-chained response sequence with the exception of the front lever response following rear response. Here, both right and left cue lights illuminated and the opposite response than the initial DSTLDD cue was required for reinforcement. Additionally, the following cue light, signaling the start of the next trial, was selected with a 50% likelihood of either left or right cue illuminating.

#### 4.5.7. Delayed Non-Match to Position Variation: DNMTP(X)

This variation of the NMTP task introduced longer delay durations between the light–dark discrimination sample response and rear cue offset, requiring the animals to maintain the front response location information longer. This delay incrementally increased for each animal from 1–25 s in 3 s intervals contingent upon achievement of 80% accuracy for two consecutive trial days during the acquisition phase. Data are presented from the intermediate 5 s delay condition to compare accuracy and rate of trial completion at a uniform time point.

#### 4.5.8. Differential Reinforcement High Rate of Response (DRH)

This task is the only operant task not based on a simple or second order conditional discrimination rule. Instead, it required multiple responses on one set lever within a 10 s response interval. Here, the required number of responses for reinforcement delivery incrementally increased by two responses upon successful completion of the response requirement of the previous session.

### 4.6. Physical and Cognitive Evaluation Procedures

The following evaluations form a comprehensive physical and cognitive battery aimed at evaluating general health and specific cognitive domains. Detailed measures, such as total distance traveled, memory errors, proportion of time dedicated to exploring, latency to escape and other parameters, were observed and recorded using the powerful tracking tool AnyMaze™.

#### 4.6.1. Paw Withdrawal Reflex

Evaluation occurred on an enclosed platform following a 5 min habituation period. The Von Frey filaments (Stoelting Co., Wood Dale, IL, USA) were pressed to front and hind paws, in ascending order, for 3 s or until retraction of the lateral plantar surface. The thickness of filaments utilized ranged 0.064–1.143mm. The smallest filament size which elicited a retraction was determined for each animal using the up-down method of testing.

#### 4.6.2. Open Field

Rats were placed in the center of a 92 cm^2^ field for 10 min while relevant measures were recorded. The total distance traveled and time in the center field assessed general exploration, mobility and anxiety-like behavior.

#### 4.6.3. Radial Arm Maze (RAM)

Rats began this task in the center circle of an 8-armed apparatus on five consecutive testing days. This study followed a 5-rewarded, 3-unrewarded version of the task, where five arms were reinforced with approximately 1 mL of a 0.2% saccharine solution. Reward locations varied within rats yet remained constant between testing days. Trials terminated upon the consumption of all five rewards or as the 10 min trial duration expired, all trials were recorded. Access to water was restricted prior to testing and free access was reinstated for a minimum of 1 h post-trial. Acquisition of this task was assessed through latency to consume all rewards across trials days, reference memory performance by entries to never reinforced arms and working memory performance by revisits to arms with previously consumed rewards.

#### 4.6.4. Novel Object Recognition (NOR)

Rats were tested in the open field apparatus over two consecutive test days. The first testing day consisted of a pre-exposure trial where animals interacted with two identical, equidistant objects for 10 min. The exposure test followed on day two. In the same open field apparatus, animals were exposed to one familiar object, from the pre-exposure day, and a novel object for 5 min. Instances of object interaction were defined as any physical contact with the object or a physical orientation towards the object within a predetermined radius of the object. Recognition memory was assessed through the number of interactions and proportion of time interacting with the novel object. All trials were recorded.

#### 4.6.5. Novel Exploration

A novel exploration task was created to examine exploration patterns when presented with multiple novel objects within a familiar context. Testing took place in the open field apparatus and animals explored four novel objects for a 5 min trial period while the number of interactions per object and proportion of time interacting with each object were measured and recorded.

#### 4.6.6. Barnes Circular Maze

Trials took place on a 125 cm diameter platform, raised 75 cm. Eight equidistant holes along the circumference of the platform served as escape or error locations. The escape box itself, measuring 31 cm × 14.5 cm × 18 cm, was attached to the underside of the platform at differing locations within rat trials, yet never differed between trial days. Rats began trials in a holding chamber centered on the platform surface to limit starting orientation bias. Trials began once the holding chamber was removed and all trials were recorded. Learning and memory were assessed by measuring of latency to escape across trial days, revisits to non-escape hole locations and path efficiency to escape (distance from start position to escape hole divided by total distance traveled to escape). Trials terminated after 5 min or upon entering the escape box.

### 4.7. Tissues Oreparation and Histopathology

Paraffin sections were cut in the sagittal plane at 20 μm thickness using a microtome. Slides were then rehydrated by immersing in xylene with decreasing concentrations of ethanol. Antigen retrieval was conducted via 5 min incubation with proteinase K (0.2 mg/mL) at 22 °C. Deposited fibrillar amyloid was detected with thioflavin-S staining, and cerebral vessels were detected with a primary antibody to collagen IV and an Alexa Fluor 594-conjugated secondary antibody. Histological images were captured on a KEYENCE BZ-X710 fluorescence microscope and analyzed with BZ-X Analyzer software.

## Figures and Tables

**Table 1:** Task summary and associated primary measures of behavioral evaluation tasks (left) and operant assessment schedules (right).

Behavioral Task	Primary Measure	Operant Schedule	Primary Measure
**Von Frey Hairs (VF)**	Limb withdrawal reflex (somatosensory-motor)	**Light/Dark Discrimination (DSTLDD)**	Discrimination learning, association of secondary reinforcer
**Open field (OF)**	General mobility	**Reaction Time (REACT)**	Motor output ability
**Novel object recognition (NOR)**	Recognition memory	**Stimulus detection (SIGDECT)**	Attention and initiation of motor output
**Novel exploration (NE)**	Interaction with spatially-arrayed stimuli	**Delayed non-matching to position (DNMTP)**	Short term memory
**Radial arm maze (RAM)**	Working and reference memory	**Differential reinforcement: high responding (DRH)**	Sustained effort, motor output
**Barnes maze**	Spatial memory		

## Supplementary Materials

Supplementary materials can be found at https://www.mdpi.com/1422-0067/21/7/2348/s1.

## Abbreviations

CAA	Cerebral amyloid angiopathy
Aβ	Amyloid β-protein
APP	Amyloid precursor protein
AD	Alzheimer’s disease
WT	Wild-type
VCID	Vascular-mediated cognitive impairment and dementia
VF	Von Frey hairs
OF	Open Field
NOR	Novel Object Recognition
RAM	Radial Arm Maze
NE	Novel Exploration
DSTLDD	Light–dark discrimination
REACT	Reaction time
SIGDECT	Stimulus detection
NMTP/DNMTP	Non-matching to position/delayed non-matching to position
DRH	Differential Reinforcement: high responding
ITI	Inter-trial interval
ANOVA	Analysis of variance
