# Supplementary material for: Longitudinal Cognitive Decline in a Novel Rodent Model of Cerebral Amyloid Angiopathy Type-1
Source: Int J Mol Sci. 2020 Mar 28;21(7):2348. doi: 10.3390/ijms21072348 (PMC7177469; doi:10.3390/ijms21072348)
Supplement: Supplementary file 1 [file ijms-21-02348-s001.pdf]

Supplementary Materials

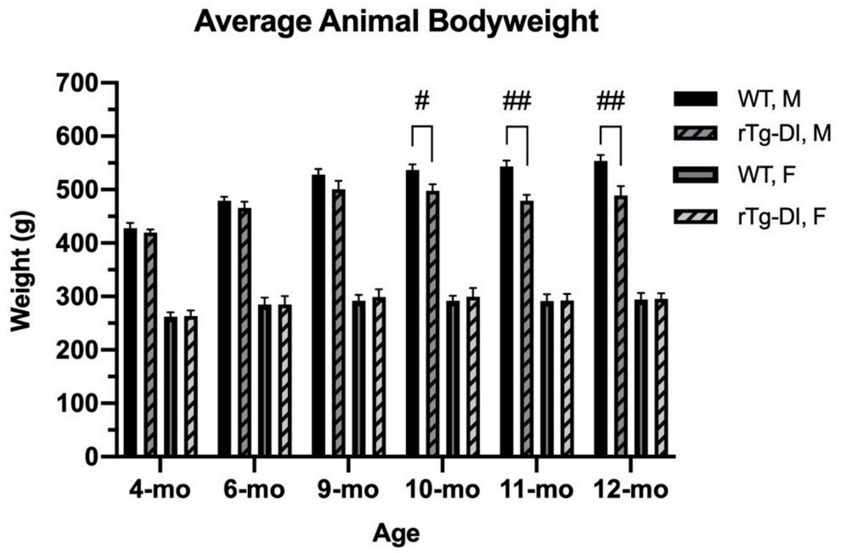

Figure 1. Average rat bodyweight. rTg-DI males weighed less at 10-, $p < .05$, 11-, $p < .005$ and 12-months, $p < .005$ than WT males. Data represent mean + SEM. *# p<.05, ## p <.005*

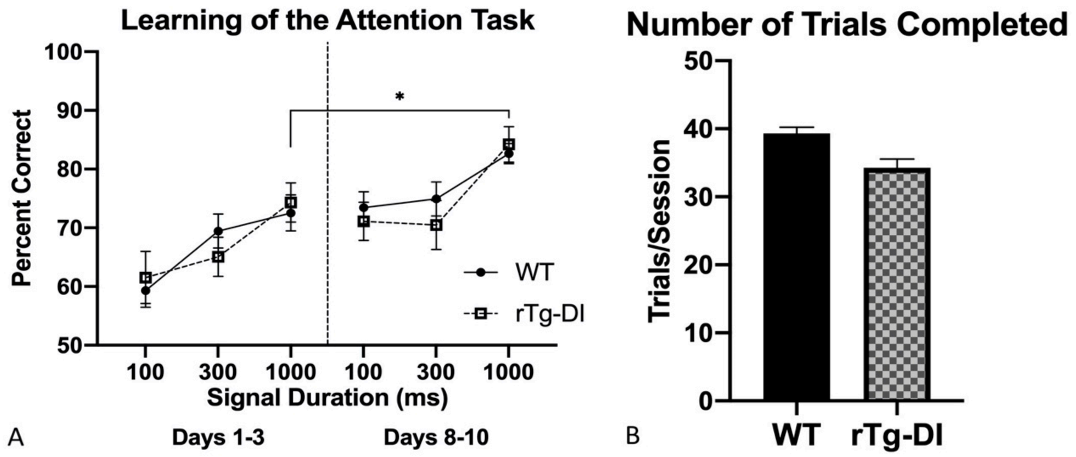

Figure 2. Signal detection and response initiation task at 7-months of age. A) Graph showing learning of signal detection and motor response; accuracy in response to the longest signal duration increased across trial days. B) The rats completed similar number of trials. Data represent mean + SEM. ** p <.01*

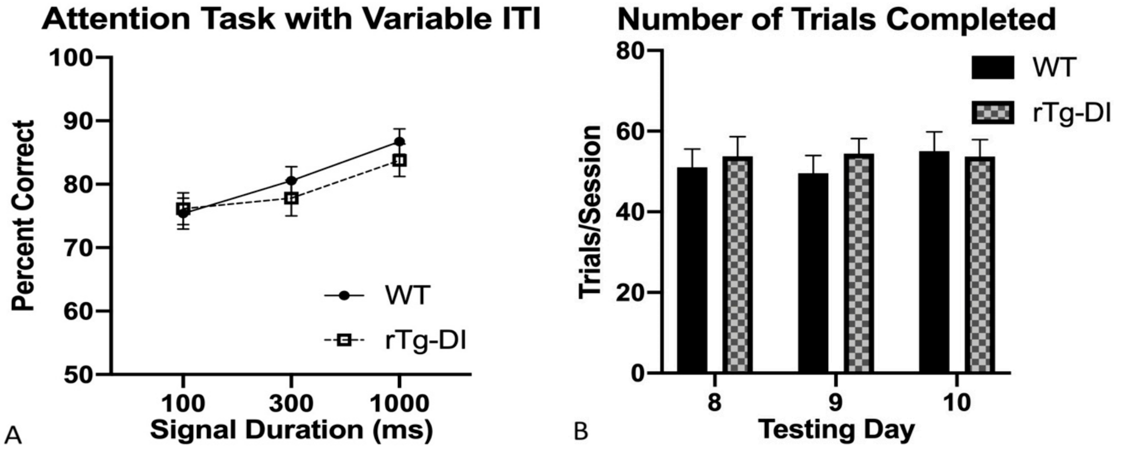

Figure 3. Signal detection with variable pre-stimulus interval task at 8-months of age. A) Graph showing learning of the varied pre-stimulus interval; rats responded accurately across trial days. B) Rats completed similar numbers of trials. Data represent mean + SEM.

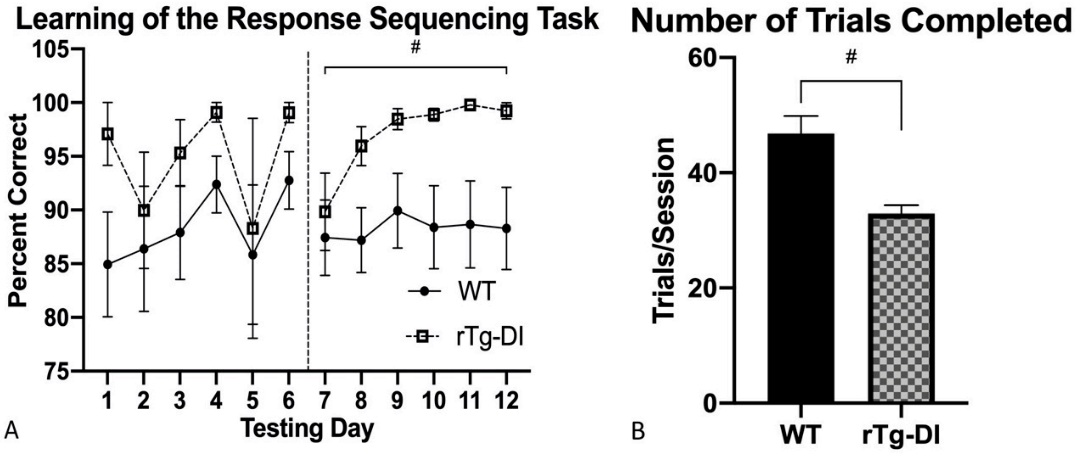

Figure 4. FR2-chained responding task at 9 months of age. A) Graph showing learning of conditional response discrimination; rTg-DI rats responded more accurately on the final trial days than WT. B) rTg-DI completed fewer trials than WT. Data represent mean + SEM. # *p<.05*

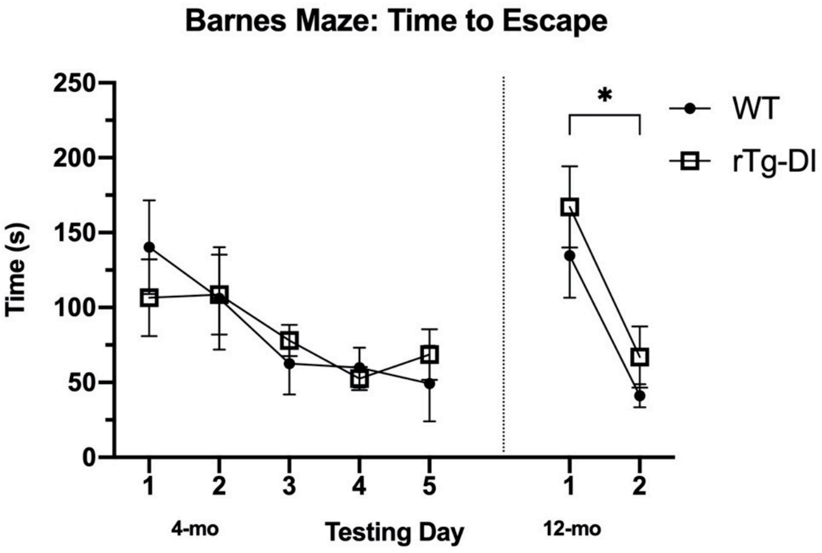

Figure 5. Barnes Circular Maze. Latency to escape; animals took a similar amount of time to escape across trial days at 4-months of age and escaped more quickly on the second trial day at 12-months than the first. * *p <.01.*

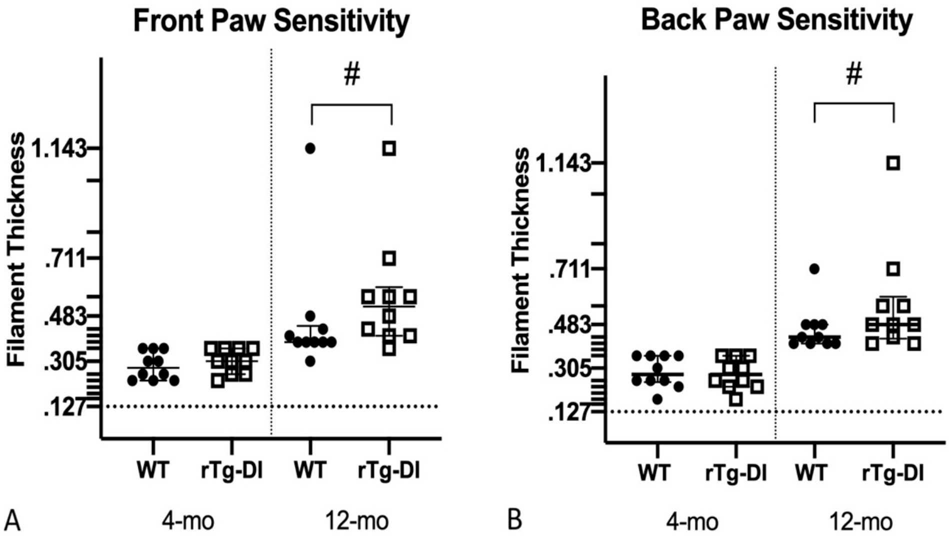

Figure 6. Paw withdrawal reflex. X-axis denotes Von Frey Hair filament thickness; the thinnest filament is represented by a horizontal dashed line. A response to a thicker filament represents less sensitivity. A) rTg-DI front paws were less sensitive at 12-months of age than WT. B) rTg-DI back paws were less sensitive at 12-months of age than WT. Data represents median with interquartile range. *# p<.05*
